# Guide to the Administration of Anæsthetics

**Published:** 1892-12

**Authors:** 


*Guide to the Administration of Anæsthetics.* By HENRY DAVIS. Second Edition. Pp. viii., 87. London: H. K. LEWIS. 1892.

The second edition of this useful little manual has been published chiefly in order to include the observations of the celebrated Hyderabad Commission, but we are happy to see that the author has not been led astray by the pernicious conclusions of that body, and still teaches what we believe to be sound doctrine on the subject of anæsthetics. We can heartily recommend it as a practical guide for students and medical practitioners.

